# Fifty Years of Research on Protonophores: Mitochondrial Uncoupling As a Basis for Therapeutic Action

**DOI:** 10.32607/actanaturae.11610

**Published:** 2022

**Authors:** E. A. Kotova, Y. N. Antonenko

**Affiliations:** Belozersky Institute of Physico-Chemical Biology, Lomonosov Moscow State University, Moscow, 119991 Russia

**Keywords:** uncouplers of oxidative phosphorylation, mitochondria, proton transport, bioenergetics

## Abstract

Protonophores are compounds capable of electrogenic transport of protons across membranes. Protonophores have been intensively studied over the past 50 years owing to their ability to uncouple oxidation and phosphorylation in mitochondria and chloroplasts. The action mechanism of classical uncouplers, such as DNP and CCCP, in mitochondria is believed to be related to their protonophoric activity; i.e., their ability to transfer protons across the lipid part of the mitochondrial membrane. Given the recently revealed deviations in the correlation between the protonophoric activity of some uncouplers and their ability to stimulate mitochondrial respiration, this review addresses the involvement of some proteins of the inner mitochondrial membrane, such as the ATP/ADP antiporter, dicarboxylate carrier, and ATPase, in the uncoupling process. However, these deviations do not contradict the Mitchell theory but point to a more complex nature of the interaction of DNP, CCCP, and other uncouplers with mitochondrial membranes. Therefore, a detailed investigation of the action mechanism of uncouplers is required for a more successful pharmacological use, including their antibacterial, antiviral, anticancer, as well as cardio-, neuro-, and nephroprotective effects.

## INTRODUCTION

The term protonophore was first used in a review by Skulachev published in 1970 [1], but protonophores were discovered several years earlier in the laboratories of Lehninger (1966 [2]), Skulachev [3], and Lieberman [4]. Those studies showed that some compounds previously identified as uncouplers of oxidative phosphorylation in mitochondria increase the proton conductivity of lipid membranes. This observation was in agreement with the Mitchell theory on the coupling of oxidation and phosphorylation in mitochondria through the electrochemical potential difference between protons [5]. In 1967, Mitchell observed proton transfer by some uncouplers in mitochondrial membranes [6]. As already mentioned, the term protonophore was coined in 1970 [1]; before that, uncouplers were called proton conductors, or H^+^ carriers [2]. It is worth noting a study in 1967 [7] on an uncoupler-mediated increase in the proton conductivity of liposomes, but that study did not attract as much research attention as the publication in Nature [3]. Skulachev’s group's priority in the discovery of protonophores was also confirmed by a publication in Nature in 1969 [8], which reported a quantitative correlation between protonophore activity in lipid membranes (planar bilayers, BLM) and stimulation of mitochondrial respiration in state 4 (P-vs-U-correlation) for many uncouplers of various chemical structures. This publication in 1969 [8] is now considered classic. It should be noted that the term ionophore, which denotes a compound that transports ions through membranes, had appeared earlier and was actively used in Pressman’s works in the mid-1960s [9]. However, Pressman focused on the transport of metal ions and did not use the term protonophore. At that time, Russian-language articles often used the term membrane-active complexone [10], which was later replaced by the term ionophore.

The listed studies caused an explosion of interest in protonophores and, together with subsequent studies, contributed significantly to proving the Mitchell chemiosmotic theory. It is worth noting that the P-vs-U correlation was immediately disputed in studies from another group [11], which reported significant deviations from the correlation for another set of compounds. Contradictions were added by Bakker et al., who showed that the P-vs-U correlation is much more stronger in liposomes than it is in planar BLMs [12]. However, the fundamental review [13] was published in 1980, which argued for the existence of a good P-vs-U correlation, while some of the contradictions were attributed to the physicochemical properties of the compounds used. Because the chemiosmotic theory was considered to have been proved by that time, the issue lost its relevance and became almost a closed one despite the fact that there was sufficient evidence of involvement of mitochondrial proteins in uncoupler effects. In particular, incubation of an azido derivative of DNP, (2-azido-4-nitrophenol (NPA), and an azido derivative of CCCP, 2-nitro-4-azidocarbonylcyanide phenylhydrazone (N3CCP), with mitochondria in response to illumination was shown to lead to covalent attachment of these compounds to a subunit of the ATPase complex [14] or a non-identified protein [15], respectively. Importantly, this covalent modification did not affect other mitochondrial proteins. But at that time, these studies were believed to contradict the Mitchell chemiosmotic theory; so they were not given sufficient attention. Interestingly, shortly after (in the 1990s), Skulachev’s laboratory published papers that pointed to the sensitivity of the DNP and CCCP effect to inhibitors acting either through specific mitochondrial proteins or through nonidentified proteins [16, 17].

## PROTONOPHORES AND LIPID MEMBRANES

**Fig. 1:**
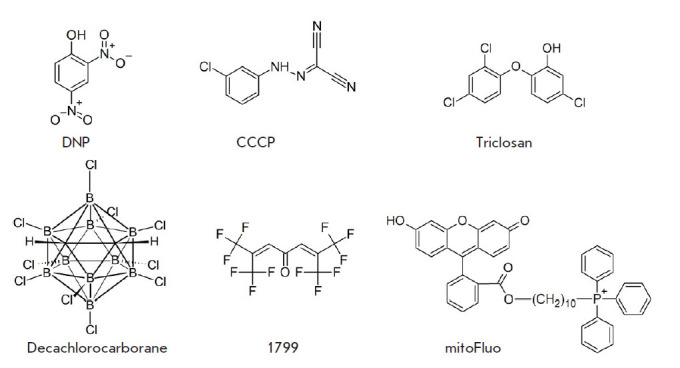
Chemical structures of conventional protonophores (top row) and unconventional protonophores (bottom row). DNP – 2,4-dinitrophenol; CCCP – carbonyl cyanide-m-chlorophenyl hydrazone; triclosan – 2,2,4’-trichloro-2’-hydroxydiphenyl ether; decachlorocarborane; 1799 – α,α’-bis(hexafluoracetonyl)acetone; mitoFluo – a conjugate of fluorescein and the triphenylphosphonium cation

Classical protonophores are organic acids with pKa close to physiological pH values, which have an extensive system of π-electrons delocalizing the negative charge that prevents penetration through the hydrophobic layer of the membrane (Fig. 1). This enables the anionic form of the protonophore (T-) to cross the membrane in response to the application of a potential, then to be protonated (transforming into the TH form), and to move in the opposite direction, as a neutral form, along the concentration gradient. The cycle is completed by deprotonation of the TH form. Apart from phenols (DNP, pentachlorophenol, etc.), various hydrazones (CCCP, FCCP), benzimidazoles (TTFB and DTFB), dicoumarol, and salicylic acid were studied among the first protonophores. These compounds, which are weak aromatic acids, correspond well to the general protonophore structure described above. However, even the first tested uncouplers included untypical examples, such as decachlorocarborane [18] and compound 1799 (α,α’-bis(hexafluoracetonyl)acetone) [11]. Strictly speaking, these compounds are not aromatic; in addition, their ability to become deprotonated in an aqueous medium also raises serious questions. Recent studies have identified cationic [19, 20, 21] and zwitterionic protonophores [22, 23, 24].

## INTERACTION BETWEEN PROTONOPHORES AND MITOCHONDRIAL MEMBRANE PROTEINS

**Fig. 2:**
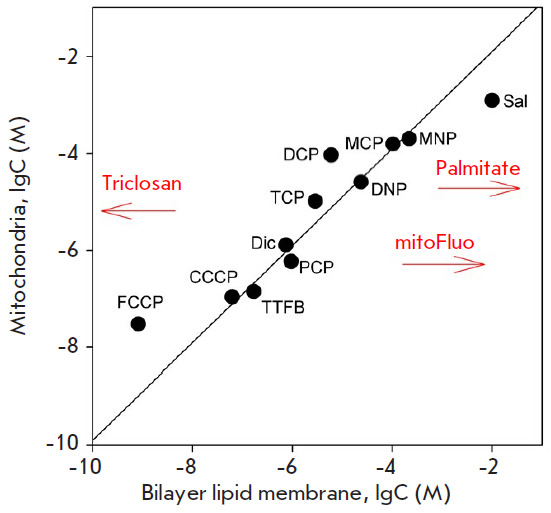
Correlation between the ability of different compounds to uncouple oxidative phosphorylation in mitochondria and their protonophoric activity in the bilayer lipid membrane (BLM) (adopted from [8]). The Y axis shows the concentrations of compounds producing a two-fold stimulation of succinate oxidation in state 4 rat liver mitochondria; the X axis shows the concentrations required to increase the conductivity of a black lipid membrane by 5×10^-9^ Ohm^-1^×cm^-2^. Red arrows mark the levels of effective concentrations of palmitate, mitoFluo, and triclosan according to [22, 40, 57]

Approximately 50 years have passed since the first studies on protonophores appeared, and many new small-molecule compounds with uncoupler properties have been identified. Many of them are described in the review [25], although the list is not complete and should be substantially expanded. Unfortunately, not all new compounds have been tested in lipid systems (BLM or liposomes), and even fewer compounds have been characterized under the same conditions. However, a lot of evidence enables significant advances in the refining of the P-vs-U correlation, compared to the first studies of the 1970s. For example, several compounds exhibiting a pronounced uncoupling effect on mitochondria but lacking protonophoric activity in lipid membranes were identified. The most known and physiologically important of these are fatty acids. It is important to emphasize that fatty acids, which increase the proton permeability of mitochondrial membranes [26, 27], have only a weak ability to increase the conductivity of planar BLMs: noticeable currents were found only in membranes formed from liposomes [28] according to the Montal method [29]. Fatty acids were shown to interact with the ADP/ATP antiporter [16, 30, 31, 32] and with other transport proteins of the SLC25 family [33], which leads to the catalysis of fatty acid anion transfer through the mitochondrial membrane. Many anti-inflammatory drugs [34] and a number of other compounds [35] have uncoupling properties. Therefore, the classical P-vs-U dependence may be significantly expanded. On the other hand, it may be concluded that the observed correlation of protonophore activity in BLMs and mitochondria is rather weak and hardly contradicts the involvement of proteins in protonophoric action in mitochondria. Figure 2 presents this correlation according to [8], with the addition of several compounds to show the magnitude of possible deviations from the canonical P-vs-U dependence (red arrows).

Compounds that effectively uncouple mitochondria but barely increase the proton conductivity of BLMs also include a recently synthesized conjugate of fluorescein and triphenylphosphonium, called mitoFluo [22]. mitoFluo has a very weak protonophore effect on BLMs, which is expected because it can be either a cation or a zwitterion. Compared to anions, cations much less efficiently penetrate BLM owing to a dipole potential, i.e., a layer of oriented dipoles at the membrane–water interface [36, 37, 38]. Zwitterions carry not only a positive charge, but also a negative one, which should further reduce their permeability. To record the mitoFluo-induced BLM current, special synthetic lipids with ether rather than ester bonds and hydrocarbon residues were used. Previously, these lipids were shown to have a significantly reduced dipole potential of the membrane [39]. Even in BLMs prepared from this lipid, mitoFluo at pH 7 did not cause a proton current; the current appeared only as pH decreased and reached a maximum at pH 3 [22]. In this case, mitoFluo, which is an effective uncoupler in mitochondria, acts at submicromolar concentrations. Another group of compounds falling out of the P-vs-U correlation includes triclosan (2,2,4’-trichloro-2’-hydroxydiphenyl ether, Fig. 2, red left arrow). Unlike fatty acids or mitoFluo, triclosan is a potent protonophore in BLMs (its effective concentrations are significantly lower compared to those of CCCP) [40]. However, triclosan is a weak uncoupler in mitochondria, and tens of micromoles of this compound are required to stimulate mitochondrial respiration [41]. Triclosan is widely used as an antimicrobial agent and is added to various cosmetic products. Its extremely weak toxicity to animal cells is associated with its weak effect on the mitochondrial membrane. The structure of triclosan suggests that it is a common anionic phenolic uncoupler, with pKa = 7.9 [42].

As mentioned above, deviations from the P-vs-U correlation are traditionally explained by the interaction between uncouplers and proteins of the inner mitochondrial membrane, which may increase proton transfer due to accelerated transfer of the anionic form of the protonophore through the lipid part of the membrane [17, 35]. This concept is well illustrated by the induction of proton conductivity in the mitochondrial membrane by fatty acids, which is significantly suppressed by the addition of carboxyatractyloside (CATR), a specific inhibitor of the adenine nucleotide translocator in mitochondria [16, 31]. Fatty acid anions are supposed to interact with the ATP and/ or ADP binding site and, thus, be transported across the membrane. High permeability of the lipid membrane for protonated fatty acids [43] enables these acids to perform the proton transfer cycle. Along with fatty acids, CATR, although to a lesser extent, inhibits the uncoupling effect of DNP in mitochondria [16, 44]. These data suggest that the DNP anion may also interact with the fatty acid binding site of the ADP/ ATP translocator. Recently, interaction between DNP and the reconstituted translocator has been shown to be blocked when arginine 79 is replaced by serine in this protein [44].

The active interaction of uncouplers with proton pumps was known even before the studies of the late 1960s–early 1970s, because all the uncouplers known at that time exhibited a bell-shaped dependence of the respiration rate of mitochondria or submitochondrial particles (SMPs) on their concentration; i.e., stimulation of respiration at low concentrations of uncouplers was always followed by its inhibition at high concentrations of uncouplers [45, 46]. This phenomenon concerns the substrates of all major mitochondrial respiratory complexes. Further, the sites and the nature of this interaction were clarified. For example, in the case of complex I, this interaction correlates well with the hydrophobicity of the compounds, which could be explained by the existence of a hydrophobic region in the protein acting as a ubiquinone binding site [47]. In succinate dehydrogenase, the most active binding site for uncouplers is the ubiquinone pocket, with its affinity for pentachlorophenol reaching 2 μM [48]. Also, cytochrome oxidase was shown to have a CCCP binding site [49], interaction with which drastically changes the protein’s affinity for oxygen [50]. Interestingly, methylation of a protonated group in uncouplers suppresses not only their uncoupling, but also their inhibitory effects [45, 51]. This important fact has not yet been explained; it indicates a close relationship between the inhibitory action and the uncoupling mechanism. It should be noted that some uncouplers are characterized by an unusually wide concentration bell [22, 52].

According to this concept, deviation of triclosan from the P-vs-U correlation in the opposite direction, compared to fatty acids, is due to the fact that most protonophores use certain proteins during the induction of proton conductivity in mitochondrial membranes. Because triclosan induces a greater BLM current than CCCP, while operating in mitochondria at larger concentrations than CCCP, the latter may be presumed to induce a proton current through some mitochondrial protein. This suggestion is supported by direct experiments on the interaction between the azido derivative of CCCP and mitochondrial proteins [15]. A recent study at our laboratory showed that the CCCP–triphenylphosphonium conjugate, which does not uncouple mitochondria, is able to block the uncoupling effect of CCCP [53]. The involvement of a protein in the uncoupling activity of CCCP is also evidenced by the strong inhibition of the CCCP effect on mitochondria by 6-ketocholestanol, which, on the contrary, can increase the CCCP-induced proton current in BLM due to an elevation of the membrane dipole potential [54]. Thus, the P-vs-U correlation in the case of conventional uncouplers is not directly related to the fact that uncouplers of oxidative phosphorylation are protonophores (i.e., proton carriers across the lipid part of the mitochondrial membrane). Apparently, this is also related to the strength of the interaction between most of these compounds and some mitochondrial protein(s).

It should also be mentioned that the P-vs-U correlation appears clearly disturbed in a series of homologues of some uncouplers. For example, our laboratory showed that the protonophoric activity of uncouplers based on the popular fluorescent dye 7-nitrobenzo-2-oxa-1,3-diazole (NBD) with an alkyl substituent grows in planar BLMs and liposomes as the alkyl chain increases [55]. In mitochondria, the uncoupling activity reaches a maximum in the case of an octyl substituent, and a decyl derivative uncouples mitochondria much more weakly than an octyl one does [55]. Similarly, in a series of alkylrhodamines (CnR1), the protonophoric activity in liposomes [56] and BLMs increases as the alkyl chain is lengthened, while maximum uncoupling in mitochondria is observed with C4R1 [21]. The optimal alkyl chain length also indicates a possible involvement of the binding sites of mitochondrial proteins in the induction of proton leakage. Of note, uncoupling by fatty acids also has an optimum for the fatty acid length: among saturated fatty acids, palmitic acid causes maximum uncoupling, whereas longer acids are less active [57]. A recent study by Samartsev’s laboratory showed that α,ω-hexadecanedioic acid stimulates mitochondrial respiration without inducing proton conductivity of the mitochondrial membrane [58]. This new phenomenon is to be studied and understood.

Thus, it may be concluded that the P-vs-U correlation is rather poor when comprising many of the new uncouplers discovered since the first studies in this field. However, it should be emphasized that the Mitchell theory, largely accepted by the scientific community owing to the P-vs-U correlation, cannot be questioned on this basis. The point is that the Mitchell theory has been proved by many direct experiments, such as the measurement of the generation of electric potentials by proton pumps [59] or the detection of ATP synthesis in liposomes with reconstructed bacteriorhodopsin and ATP synthase [60]. In addition, there is no doubt that the uncoupling effect of gramicidin A is mediated by the formation of a proton channel and induction of proton leakage in the inner mitochondrial membrane. The Mitchell theory puts emphasis not on the P-vs-U correlation but on the correlation between mitochondrial uncoupling (i.e., stimulation of respiration and ATP hydrolysis) and the protonophore activity of uncouplers, which is measured directly in mitochondria [61]. In the Mitchell theory, it is not important whether the uncoupler induces a proton current in the mitochondrial membrane via the lipid parts of the membrane or via some mitochondrial protein. Proton leakage in the mitochondrial membrane may be measured under deenergized conditions based on the swelling of mitochondria in a medium with potassium acetate in the presence of valinomycin or with ammonium nitrate without valinomycin [26]. This technique was used to show that fatty acids induce proton conductivity in the inner mitochondrial membrane at the same concentrations at which they stimulate mitochondrial respiration [26]. Thus, despite the fact that fatty acids fall out of the P-vs-U correlation, their induction of proton conduction in mitochondria only confirms the Mitchell theory.

Another question is the existence of a protonophore that acts in mitochondria without the involvement of proteins. As described above, the most popular uncouplers DNP and CCCP may hardly be considered such protonophores. Gramicidin A may be such a protonophore, but it transports not only protons, but also potassium and sodium ions, which makes it very toxic to cells. Perhaps, this role may be played by triclosan, an extremely active protonophore in BLMs, surpassing both CCCP and SF6847, the most potent known uncoupler [40]. However, triclosan causes a stimulation of mitochondrial respiration and their swelling in a medium with potassium acetate (in the presence of valinomycin) only at a concentration of 3–10 µM. Thus, triclosan strongly deviates from the P-vs-U correlation (Fig. 2, red arrow on the left). According to [40], this deviation from the P-vs-U correlation may be caused by the high hydrophobicity of triclosan, which complicates the penetration through the outer mitochondrial membrane. However, even this weak uncoupling activity may be due to the interaction of triclosan with some protein. In this regard, it should be mentioned that triclosan interacts with mitochondrial NADH dehydrogenase and inhibits it at higher concentrations (30–100 μM) [41].

## PROTONOPHORES AND PROTON PUMPS

**Fig. 3:**
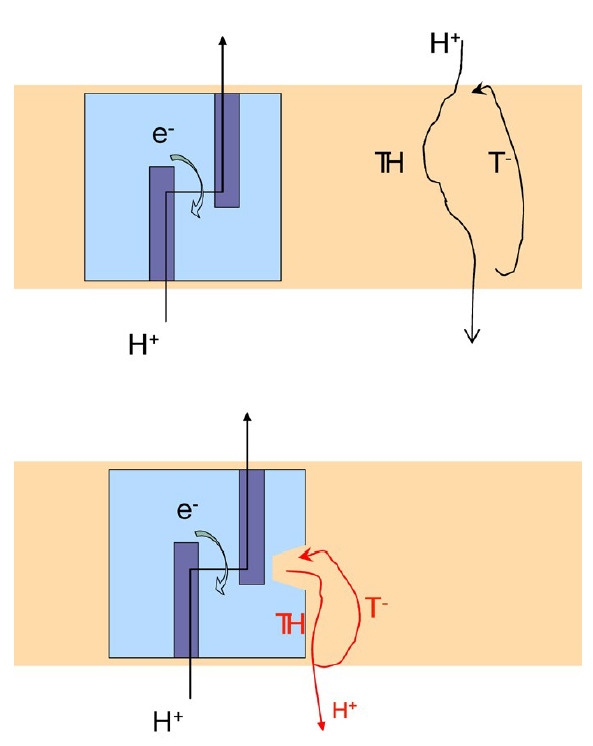
Schematic of the protonophoric effect of an anionic uncoupler T (top) and a modified model of direct interaction between T and the proton channel of the proton pump (bottom). The protonophore T transfers protons as a protonated complex TH and comes back as an anionic form T^-^ via the deprotonation cycle at the membrane interface

Above, we considered the mechanism of interaction between DNP and the ATP/ADP translocator, which contributes to the uncoupling effect of DNP on mitochondria [44]. According to our data, the translocator is also involved in the uncoupling effect of a new popular uncoupler BAM15 [62]. However, there may be also a universal mechanism of interaction between uncouplers and mitochondria, which differs from the direct proton transfer across the lipid part of the membrane. The following action mechanism of uncouplers may be proposed, which, on one hand, involves the ability to transfer protons across the lipid part of the membrane and, on the other hand, explicitly requires their interaction with proton pumps. This mechanism may be characterized as capture ("stealing") of protons from the proton pump channels (lower diagram in Fig. 3). All proton pumps are known to have proton channels that are lined with appropriate amino acids to protect the proton from leakage into the aqueous phase. But nature did not need to protect the proton pathways from leakage into the lipid phase, because the hydrated proton is very hydrophilic, and there is a huge energy barrier to its transition into the lipid phase. Therefore, some channels of proton pumps (probably, most of these channels) may lack complete isolation from proton leakage in the hydrophobic layer of the membrane. Because protonophores are lipophilic acids, they are able to intercept the protons that are pumped out of the mitochondrial matrix during the transfer of electrons along the respiratory chain and return them to the matrix, even before they enter the intermembrane space. This causes an abortive proton cycle which is similar to classical uncoupling. This idea is consistent with a previously proposed mechanism of proton slips in proton pumps [63], which was discussed in connection with distortions of the membrane integrity caused by organic solvents or other rough effects. In addition, this concept explains the suppression of proton pumps at high concentrations of uncouplers because interaction with the proton channel of the mitochondrial pump at an increased concentration may lead to complete blocking of this channel, thereby causing inhibition of the enzyme. Because the structures of most mitochondrial proton pumps have already been established, a hypothesis of the mechanism of mitochondrial uncoupling may be tested using a bioinformatics analysis. Further research will show the validity of this hypothesis.

## PROTONOPHORES AND MILD UNCOUPLING

Although the term protonophore is defined quite clearly (a protonophore is capable of electrogenically transferring a hydrogen cation through a hydrophobic phase), the use of this term for mitochondria encounters certain difficulties when combined with the term uncoupler. For example, should induction of leaks caused by detergents [64, 65, 66] or organic solvents [63] be called a protonophoric effect? In this case, a proton leak is also induced, but because there are leaks of other ions, it is hardly sensible to call this a protonophore effect. The question of whether penetrating organic cations accumulating in mitochondria, such as mitoQ and SkQ, are protonophores is more complicated. These cations are able to transport fatty acid anions across membranes and act as inducers of proton conductivity of the membranes in the presence of fatty acids, which are usually present in cells [67]. There are articles where the term protonophore is applied to mitoQ [25] and SkQ [68]. However, these cations are not capable of transporting protons across membranes; therefore, the term protonophore is not appropriate for them. On the other hand, they may be called uncouplers.

Another, rather controversial, concept associated with the use of uncouplers is the term "mild uncoupling". This term was proposed by Skulachev [17] and Starkov [69] to denote the mitochondrial state that is characterized by a reduced membrane potential, a reduced generation of reactive oxygen species (ROS), weak stimulation of respiration, and persistent high activity of ATP synthase. This state may be induced by mechanisms inherent to mitochondria (uncoupling by endogenous fatty acids or UCP family proteins) or by the addition of a small concentration of uncouplers. The term mild uncoupling was introduced in connection with the discovery of a nonlinear dependence of ROS generation on the mitochondrial membrane potential [70]. Although the concept of mild uncoupling has not been quantified, it may be considered appropriate due to numerous examples of the therapeutic effect of low uncoupler concentrations in physiological models of various pathological conditions [71]. We will consider this issue in more detail when discussing the therapeutic effect of uncouplers.

## APPLICATIONS OF PROTONOPHORES

The history of the investigation of protonophores dates back more than 50 years. In conclusion of our brief review, we would like to consider the practical application of protonophores. We should start with the history of DNP that was used as a remedy for obesity in the 1930s [72]. This was an over-the-counter drug that was used by more than 100,000 people, but it was prohibited in 1938 due to the side effects associated with hepatotoxicity and vision problems. Now, interest in DNP has re-emerged [73] due to the appearance of more complex DNP forms, such as ethyl ethers [74], which are converted into DNP mainly in the liver, or DNP complexes with nanoparticles [75]. These drugs show strong anti-diabetic activity in rats and are also effective against a non-alcoholic fatty liver disease. The clinical fate of protonophores, which are used as anthelmintic drugs, is more successful. These include salicylanilides: e.g., niclosamide. The action mechanism of these drugs is defined as the uncoupling of oxidative phosphorylation in worm cells [76, 77]. However, they have little effect on the human body because they are poorly absorbed in the gastrointestinal tract. Many protonophores also exhibit antimicrobial activity [78]. However, their general toxicity precludes their use as antibiotics. Strong protonophores such as triclosan, usnic acid [51], niclosamide [79], and pyrrolomycin [80] exhibit only a moderate toxic effect on eukaryotic cells with a very strong antimicrobial effect. Some anti-tuberculosis drugs also have a protonophoric effect [81, 82, 83]; usnic acid also has an anti-tuberculosis effect. In general, protonophores remain relevant for pharmacology and in some areas their potential is even growing.

We may also mention the insecticidal, herbicidal (pesticidal), and fungicidal effect of protonophores: dinitrophenol analogs, such as pentachlorophenol [84], 6-isobutyl-2,4-dinitrophenol (dinoseb) [85], fluazinam [86, 87], etc. We are talking about a fairly large production and a market for agriculture and the forestry industry (wood preservatives). However, in this review, of great importance is not the industrial application of protonophores but their potential significance for pharmacology. After many years of studying protonophores, a lot of data about their protective properties have been collected through animal disease models: they may be used as cardioprotectors [88], neuroprotectors [73, 89], nephroprotectors [90], radioprotectors [91], and exhibit antidiabetic activities [75, 92, 93], and the list goes on. Uncouplers may be used as anticancer agents [94]. Furthermore, low doses of DNP significantly increase the lifespan of rats [95], yeasts [96], and fruit flies [97]. As mentioned above, this protective effect is due to the ability of uncouplers to suppress the formation of ROS in mitochondria, which is largely controlled by the membrane potential [98]. Recent studies suggest that a decrease in the mitochondrial membrane potential in cells due to low concentrations of uncouplers may trigger a whole cascade of changes in the cell metabolism, which may lead to an increase in the mitochondrial mass in some cells [99, 100], activation of mitophagy [101], changes in the ratio of glycolysis to oxidative phosphorylation [102], and many others [89, 103]. The important role of calcium and cAMP in the alteration of cell metabolism is confirmed by the results of many studies [73, 100, 101, 102, 103]. Gao et al. suggested that mild uncoupling may be used to call such a state where the dose of a used uncoupler does not lead to a decrease in the proliferative potential of cells but significantly affects some regulatory cascades, such as STAT3 [104].

Thus, a detailed study of the action mechanism of protonophores in mitochondria remains an important problem. Its solution may help towards a switch from animal experiments to the use of protonophores in clinical practice, not only as anthelmintic agents, but also as drugs effective against various common and severe diseases.

## Abbreviations

DNP: 2,4-dinitrophenol
CCCP: carbonyl cyanide-m-chlorophenylhydrazone
BLM: bilayer lipid membrane
P-vs-U correlation: correlation of uncoupling efficiency in mitochondria and protonophoric activity in bilayer lipid membranes
CATR: carboxyatractyloside
mitoFluo: triphenyl-phosphonium cation fluorescein conjugate
